# Research Priorities on One Health: A Bibliometric Analysis

**DOI:** 10.3389/fpubh.2022.889854

**Published:** 2022-05-31

**Authors:** Liyuan Miao, Hao Li, Wei Ding, Shenning Lu, Shuning Pan, Xiaokui Guo, XiaoNong Zhou, Duoquan Wang

**Affiliations:** ^1^NHC Key Laboratory of Parasite and Vector Biology, School of Global Health, Chinese Center for Tropical Diseases Research, Shanghai Jiao Tong University School of Medicine, Shanghai, China; ^2^National Institute of Parasitic Diseases, Chinese Center for Disease Control and Prevention (Chinese Center for Tropical Diseases Research), Shanghai, China; ^3^WHO Collaborating Centre for Tropical Diseases, Shanghai, China; ^4^National Center for International Research on Tropical Diseases, Shanghai, China; ^5^One Health Center, Shanghai Jiao Tong University-The University of Edinburgh, Shanghai, China; ^6^School of Public Health/Global Health Institute, Wuhan University, Wuhan, China; ^7^Center for Social Sciences and Institute for Advanced Studies in Social Sciences, Southern University of Science and Technology, Shenzhen, China

**Keywords:** One Health, bibliometric analysis, research priority, research status, research hotspots

## Abstract

**Objective:**

One Health is an emerging research area that has received increasing attention globally. In this study, we aimed to explore the global research trend and hotspots of One Health and provide a reference for potential future research and practices.

**Methods:**

This was a bibliometric descriptive study of publications on One Health in four directions, including zoonotic diseases, antimicrobial resistance, food safety, and vector-borne infections. Publications from 2003 to 2021 were retrieved using the Scopus database on One Health, which were screened based on the PRISMA guidelines. Keywords were analyzed and visualized using VOSviewer software.

**Results:**

A total of 12,815 publications were included. The annual number of publications and those on each topic showed a gradual increase from 181 in 2003 to 1,647 in 2020, with an average annual growth rate of about 20.2%; the top three countries in terms of the number of publications were the United States of America (n=3,588), the United Kingdom (n=1,429) and China (n=1,233); the major research subjects were mainly in the natural sciences, with fewer social sciences subjects involved (*n* = 312; 1%). The main research directions within the area of zoonotic diseases included viral, bacterial, parasitic zoonotic diseases, and vector-borne diseases, with a small amount of antimicrobial resistance research. The major research interests within antimicrobial resistance were *Enterobacteriaceae* drug-resistant bacteria, methicillin-resistant *Staphylococcus aureus, Pseudomonas aeruginosa*, and antimicrobial resistance gene detection; research on food safety clustered around agronomy research, aquaculture research as well as a small amount of antimicrobial resistance research in food; and research on vector-borne diseases focused on mosquito-borne infectious diseases, tick-borne infectious diseases, and vectors.

**Conclusions:**

The scientific literature on One Health has witnessed a rising global trend. Most research has focused on the human-animal health interface, while environmental health is often neglected. Research subjects mainly fall within natural science disciplines, with less social science research. More support needs to be given to interdisciplinary and intersectoral cooperation and research in the future.

## Introduction

Continuous outbreaks of emerging and re-emerging infectious diseases are now rampant, threatening human health and wreaking havoc on socioeconomic development (1). The World Health Organization (WHO) stated in its 1996 World Health Report that “we are on the brink of a global crisis of infectious diseases from which no country is safe from infectious diseases.” The COVID-19 pandemic is affected by many factors with the globalization, such as mobile populations, rapid circulation of animals and food, international trade, lifestyle changes, nutritional conditions, and changes in the ecological environment, among others. The COVID-19 pandemic proves once again that an outbreak is only one flight away. Emerging infectious diseases are dominated by zoonoses, with more than 70% of them associated with or originating from wildlife (2). Many countries are experiencing biodiversity loss due to habitat alteration primarily related to deforestation or forest degradation, as well as factors such as climate change and overexploitation of natural resources. This may lead to the re-emergence of diseases that have remained undetected for a long time (3), such as Sabiá virus that re-emerged in 2020 in Brazil (4), Rocio virus in 2011 in Brazil (5) and Machupo virus in 1990 in Bolivia (6). We are facing more complex health challenges than ever before, which compels a focus on public health (7). No single discipline, institution, organization, or country can address today's complex and multifaceted global health issues. Instead, it takes cross-regional, multisectoral, and multidisciplinary approaches to combat emerging and re-emerging infectious diseases (8).

The discipline of global health focuses on human health and aims to achieve health equity on a global scale, but this is not enough to address complex health issues; it requires the integration of people, animals, and the environment, which gives rise to the concept of One Health. The One Health High-Level Expert Panel (OHHLEP) defined One Health as follows: One *Health is an integrated, unifying approach that aims to sustainably balance and optimize the health of people, animals, and ecosystems. It recognizes that the health of humans, domestic and wild animals, plants, and the wider environment (including ecosystems) are closely linked and interdependent. The approach mobilizes multiple sectors, disciplines, and communities at various levels of society to work together to foster well-being and tackle threats to health and ecosystems while addressing the collective need for clean water, energy, and air, safe and nutritious food, taking action on climate change, and contributing to sustainable development* (9). In recent years, the One Health concept has gradually gained traction in public health and animal health communities. To date, many countries have actively applied the One Health approach to public health, international health, and global health governance processes. Since One Health is an emerging discipline, extensive literature research is required to summarize past research hotspots and guide subsequent in-depth studies and practices.

Bibliometrics is a statistical method used to analyze a body of literatures and its bibliometric characteristics, evaluate the development of a specific field, and predict its future trends (10). In particular, this method allows for a presentation of the knowledge structure and evolution of a research area through mathematical statistics and visual knowledge mapping. The results offer a panorama of the field as well as reveal the trending topics to help establish further research directions (11). In this study, we conducted a bibliometric analysis of the publications on One Health from 2003 to 2020 from Scopus core collection database with the aim of revealing the global research trend of One Health and providing a reference for potential future research and practices.

## Methods

### Study Design

Following the PRISMA guidelines, this study used the bibliometric approach to analyze the research trends on One Health by (a) time and number of publications; (b) countries of publications; (c) most active journals; (d) subject areas; (e) citation of publications; (f) keywords of publications.

### Search Strategy

We used the Scopus database as the data source for this study. Scopus is one of the largest bibliometric database that contains the metadata necessary for a bibliometric analysis (12, 13) and combines the features of PubMed and Web of Science with a broader range of journals. Based on previous bibliometric analyses, Scopus is the appropriate data source (14, 15).

According to the Centers for Disease Control and Prevention (CDC), zoonotic diseases, antimicrobial resistance, food safety, and vector-borne infections are the four priority topics of One Health (16). Accordingly, we set the search formula as TITLE-ABS-KEY [human AND (animal OR wildlife) AND (environment OR plant OR eco^*^)] AND TITLE-ABS-KEY (“zoonotic” OR “zoonosis” OR “antimicrobial resistan^*^” OR “antibiotic resistan^*^” OR “anti-bacterial resistan^*^” OR “food safety” OR “food security” OR “vector-borne”). The retrieved publications represent research conducted at the human-animal-environment interface on the four priority topics of One Health.

### Data Collection and Processing

**Figure 1:**
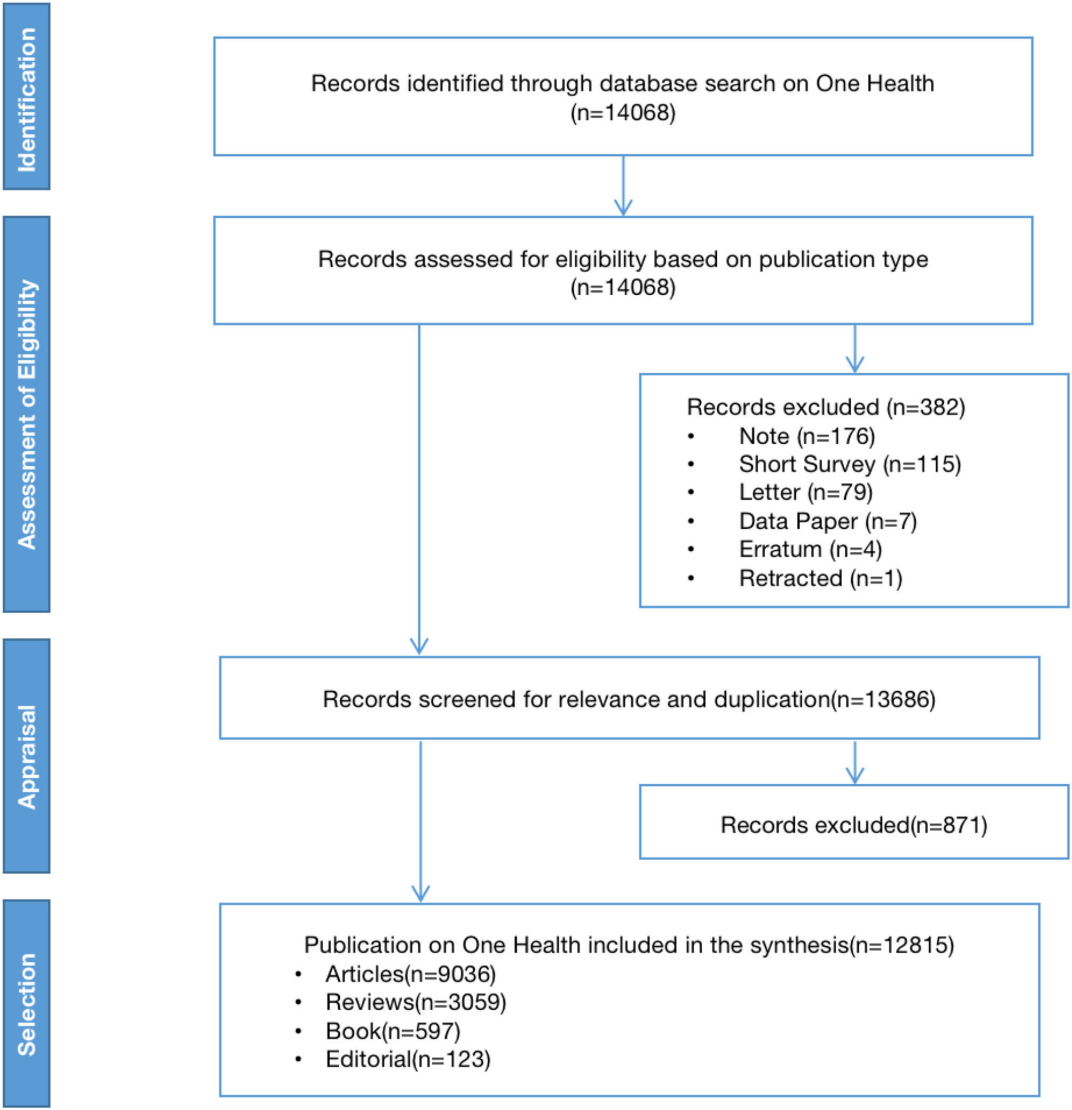
1PRISMA framework.

The term “One Health” was first coined in 2003 by William Karesh, DVM (17). Hence, we used April 2003 as the starting point for the search. The search period was April 2003-October 2021 (retrieved on October 14, 2021). According to the inclusion and exclusion criteria, 12,815 publications were finally obtained (Figure 1).

In the Scopus database, the Full Record and Cited References were exported in 7 steps of with 2,000 records each and imported into VOSviewer 1.6.15. In the software, we set the Type of analysis as “Co-occurrence,” the Unit of analysis as “Author keywords,” and the threshold value as 10 to draw the keyword co-occurrence clustering analysis map.

### Data Analysis

In this study, we used VOSviewer, a visualization tool, to perform keyword clustering analysis on the collected literature. The software constructs and presents bibliometric maps with differences in distance, size, and density between references based on the co-citation principle of literature, which can be used to form a clustered view of the literature to assess its research directions and hotspots (18). Clustering is the grouping of data objects into multiple classes or clusters, the division of which is based on the principle that there is a good similarity among objects in the same cluster, while objects in different clusters are more different from each other (19). Keywords of the same color belong to the same cluster, and different colors distinguish different clusters. The size of the circle represents the number of occurrences, and a higher density of clusters means that they are more closely connected and relevant to each other (20). Although there is no clear relationship between keywords appearing in the same publication, we can still infer the relationship between these keywords by analyzing the specifics of their occurrence. By analyzing the co-occurrence clustering of high-frequency subject words or noun phrases in a specific field or discipline, we can objectively chart the current research hotspots in the One Health field (21).

## Results

### Number of Publications

**Figure 2:**
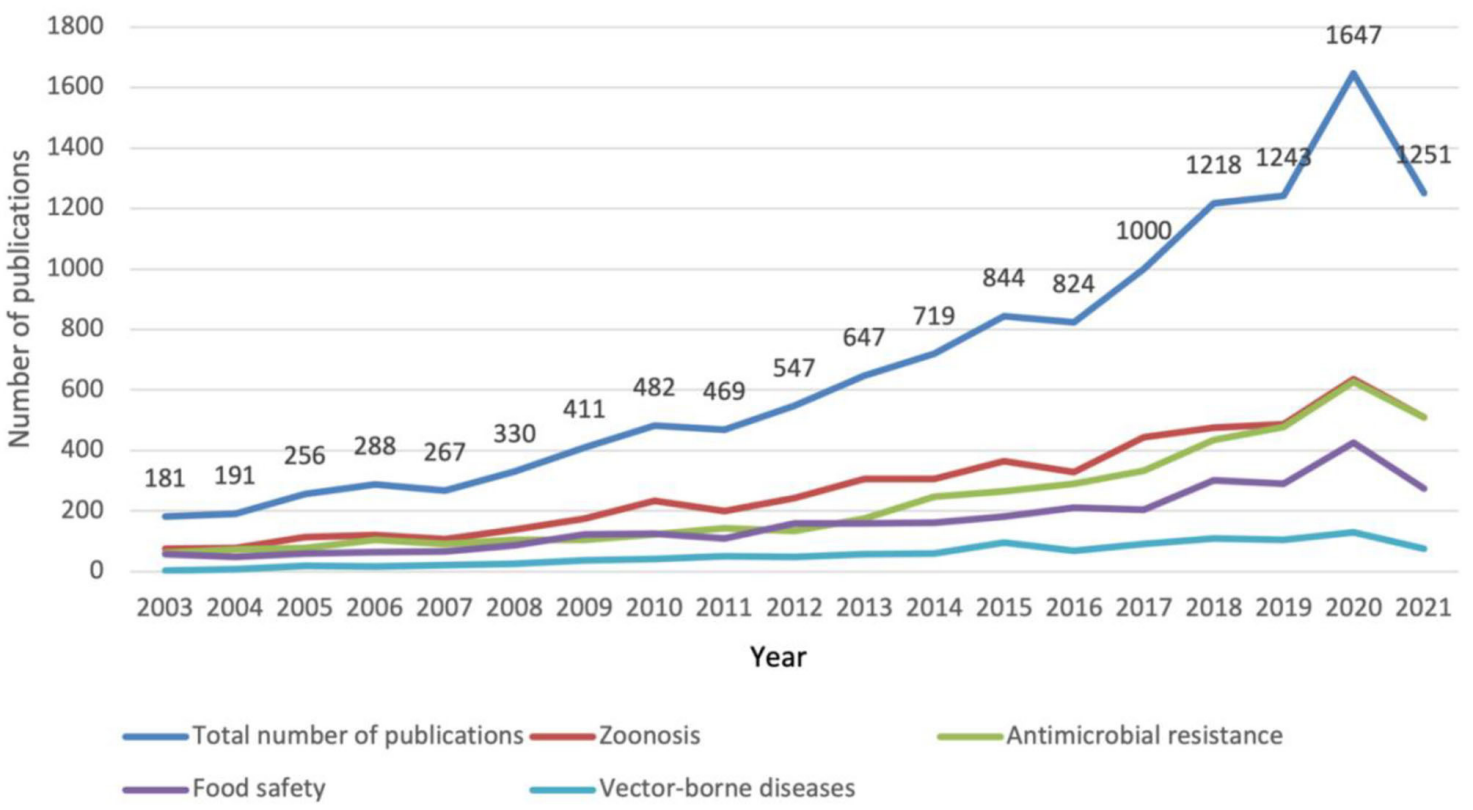
Number of publications on the four priority topics of One Health, 2003–2021.

Overall, the number of publications related to the four research topics on One Health has steadily increased over the past 18 years with varying rates among years (Figure 2). The number of publications showed a slow growth from 181 to 267 in the years 2003-2007, which is followed by a significant rise from 330 to 844 between 2008 and 2015. Growth accelerated again between 2016 and 2019. By 2020, the figure has hit 1,647, which is 9 times more than the number in 2003, with an average annual growth rate of about 20.2%. As of October 14, 2021, the number of publications in 2021 has reached 1,251.

Among the four topics, the number of zoonosis-related publications has been on a steady rise; the growth of antimicrobial resistance publications has accelerated since 2014 and has reached the same level as zoonosis publications in 2019; the number of food safety publications has only started to show an accelerated growth in 2017, while the number of vector-borne disease publications has remained in a slow-growth phase with a low number of publications.

### The Most Productive Countries

**Figure 3:**
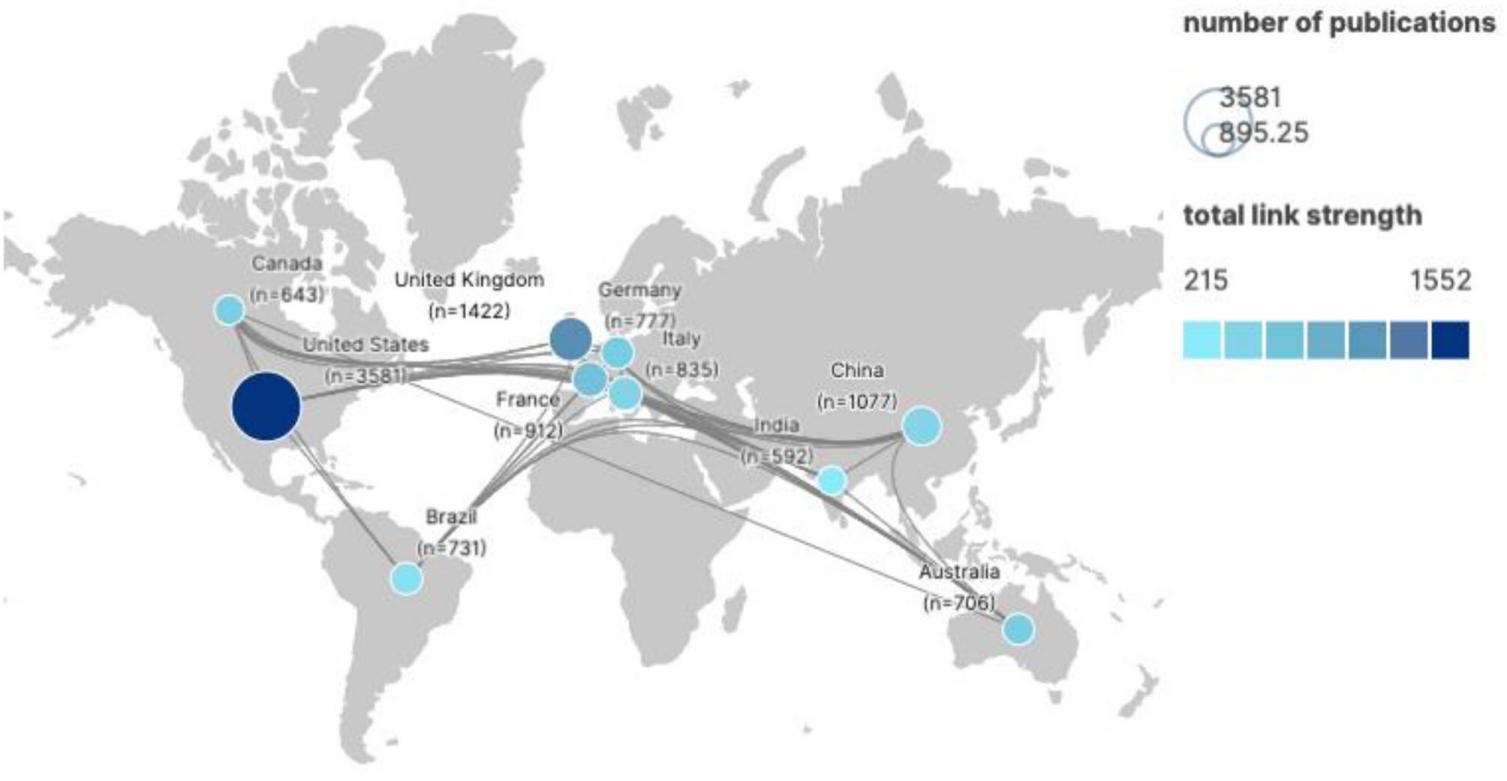
Top 10 national collaborative networks for the four priority topics of One Health, 2003–2021.

The 10 countries producing the highest number of papers on One Health are the United States of America (USA), United Kingdom (UK), China, France, Italy, Germany, Brazil, Australia, Canada, and India (Figure 3). The US produced the most publications (3,581; 27.94%), ranking in the top tier of this field. The UK (1,422) and China (1,077) rank in the second tier. The third tier includes France, Italy, Germany, Brazil, Australia, Canada, and India, all with more than 500 publications. In terms of cooperation, the US ranks first with 1,552 total link strengths. China, in contrast, has less cooperation with other countries despite its high number of publications, with a total link strength of 482, ranking eighth.

### The Most Active Journals

The top 10 active journals are listed in Table 1. These journals published 1,731 articles in total (13.5% of all articles published on One Health). *PLoS ONE* was the most prolific journal with 343 publications followed by *PLoS Neglected Tropical Diseases* with 212 publications. The top 10 active journals included three journals in the field of zoonoses, three in the field of environment, and two in the field of vector-borne diseases. *PLoS ONE* received the highest normalized citation per publication. Half of the journals in the top 10 list were ranked by Scopus as Q1 and all of them were affiliated with countries in the top 10 list for the number of publications, mainly with the US, UK, Switzerland, and the Netherlands. In addition, two specialized journals - *One Health* and *International Journal of One Health*, did not rank in the top 10 due to the smaller number of publications.

**Table 1:** Active journals in four priority areas of One Health research, 2003–2021.

**Rank**	**Journal**	**Frequency**	**Citations**	**Rank**	**Affiliation**
1	PLoS ONE	343	9188	Q2	USA
2	PLoS Neglected Tropical Diseases	212	5293	Q1	USA
3	Frontiers in Microbiology	175	5056	Q1	Switzerland
4	Zoonoses and Public Health	170	3387	Q3	UK
5	Science of the Total Environment	168	5580	Q1	Netherlands
6	Parasites & Vectors	157	4167	Q1	UK
7	Vector-Borne and Zoonotic Diseases	135	3107	Q3	USA
8	Acta Tropica	125	2832	Q2	Netherlands
9	International Journal of Environmental Research and Public Health	124	2198	Q2	Switzerland
10	Applied and Environmental Microbiology	122	6015	Q1	USA
	One Health			Q2	Netherlands
	International Journal of One Health				India

### The Most Prolific Authors

Table 2 lists 21 prolific authors contributing 21 or more papers during the study period. Of the 21 prolific authors, 5 were from the US, 3 from Portugal, 3 from Belgium and the remaining 10 from the UK (2), Spain (2), France (2), Italy, India, Germany and Denmark. Two of the 21 prolific authors were from Universiteit Gent (Belgium) and the remaining 19 authors were scattered in 19 different institutions. These authors published 539 (4.2 %) publications. Delia Grace of the Natural Resources Institute, University of Greenwich (UK) topped the list with 40 papers.

**Table 2:** Prolific authors in four priority areas of One Health research, 2003–2021.

**Rank**	**Author**	**Institution**	**Frequency**
1	Delia Grace	Natural Resources Institute, University of Greenwich, UK	40
2	Domenico Otranto	Università degli studi di Bari Aldo Moro, Italy	36
2	Carmen Torres	Universidad de La Rioja, Spain	36
3	Peter Daszak	EcoHealth Alliance, USA	33
4	Gilberto Igrejas	University of Trás-os-Montes and Alto Douro, Portugal	27
5	Kuldeep Dhama	Indian Veterinary Research Institute, India	26
5	Luísa Peixe	Universidade do Porto, Portugal	26
6	William B Karesh	Cuyahoga County Board of Health, USA	25
6	Patrícia Poeta	Centro de Ciência Animal e Veterinária, Portugal	25
7	Richard S Ostfeld	Institute of Ecosystem Studies, USA	24
8	Teresa M Copue	CIBER in Infectious Diseases (CIBERINFEC), Spain	23
8	Jean Yves Madec	Université de Lyon, France	23
8	Jonna A K Mazet	University of California, Davis, USA	23
8	Jonathan R Rushton	University of Liverpool, UK	22
9	Pierre Dorny	Prins Leopold Instituut voor Tropische Geneeskunde, Belgium	22
9	Mendel Friedman	USDA ARS Western Regional Research Center (WRRC), USA	22
9	Stefan Schwarz	Freie Universität Berlin, Germany	22
10	Anders Dalsgaard	Københavns Universitet, Denmark	21
10	Brecht Devleesschauwer	Universiteit Gent, Belgium	21
10	Sarah Gabriël	Universiteit Gent, Belgium	21
10	Serge Morand	CNRS Center National de la Recherche Scientifique, France	21

### Research Subjects

**Figure 4:**
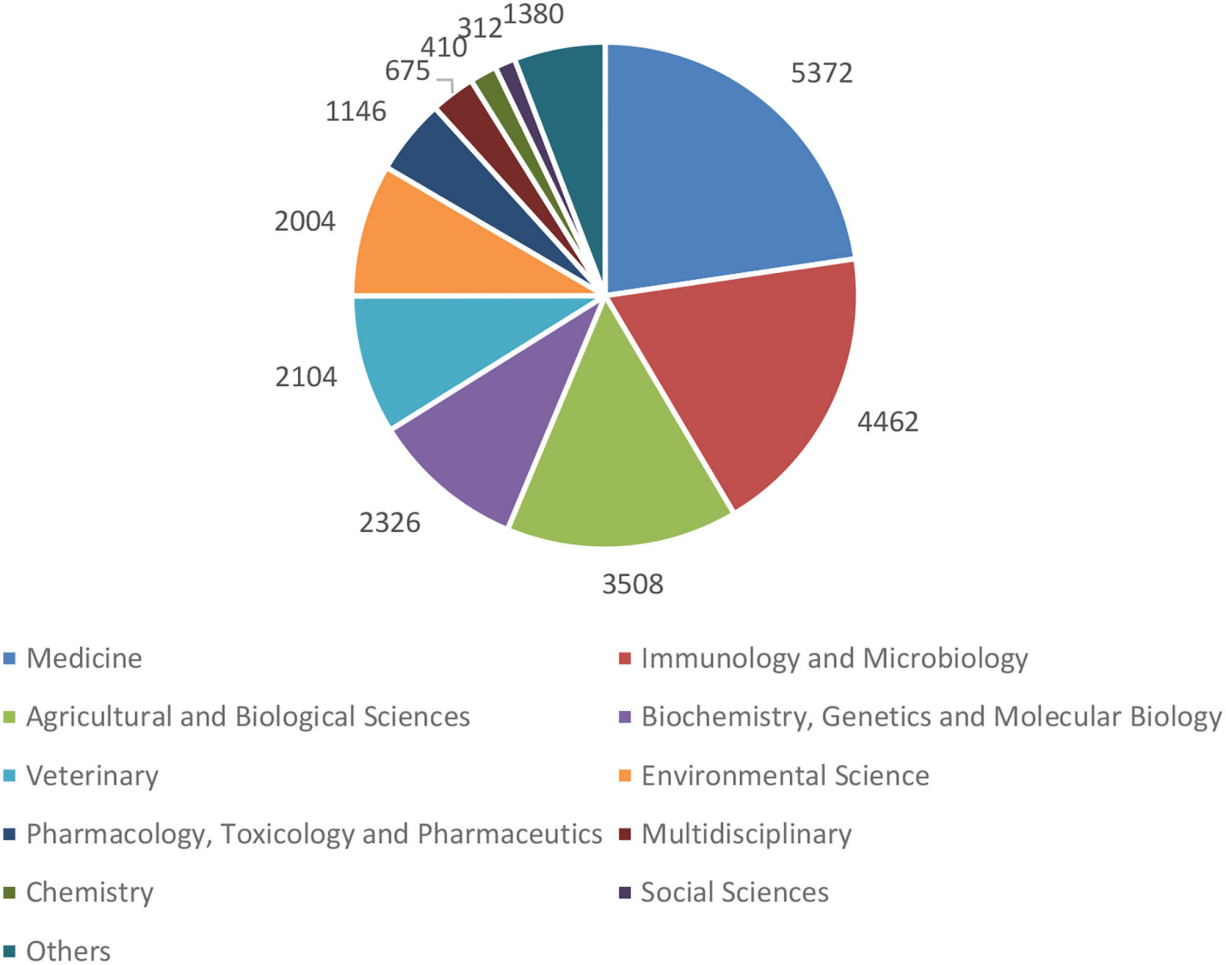
Subject distribution of the four priority topics of One Health, 2003–2021.

Figure 4 shows the subject distribution of the publications on the four priority topics of One Health. The top 10 subjects are medicine, immunology and microbiology, agricultural and biological sciences, biochemistry, genetics and molecular biology, veterinary, environmental sciences, pharmacology, toxicology, and pharmaceutics, multidisciplinary, chemistry, and social sciences. Most of the top 10 subjects are in the natural sciences. “Others” contain subjects such as engineering, nursing, and economics.

### The Most Cited Publications

The top 10 cited papers were listed based on Scopus databases. Among the top 10 cited publications, most were published between 2004 and 2015 on antimicrobial resistance (7) (Table 3).

**Table 3:** Top 10 cited One Health publications in the Scopus database, 2003–2021.

**Rank**	**Title**	**First author(s)**	**Corresponding author(s)**	**Journal**	**Citations**	**Year**
1	Global trends in emerging infectious diseases	Jones KE	Daszak P	Nature	3843	2008
2	Global pollinator declines: Trends, impacts and drivers	Potts SG	Potts SG	Trends in Ecology and Evolution	3054	2010
3	Antibacterial resistance worldwide: Causes, challenges and responses	Levy SB, Bonnie M	Levy SB, Bonnie M	Nature Medicine	2486	2004
4	A global perspective on the use, sales, exposure pathways, occurrence, fate and effects of veterinary antibiotics (Vas) in the environment	Sarmah AK	Sarmah AK	Chemosphere	2274	2006
5	Antibiotic resistance-the need for global solutions	Laxminarayan R	Cars O	The Lancet Infectious Diseases	2151	2013
6	Comprehensive evaluation of antibiotics emission and fate in the river basins of China: Source analysis, multimedia modeling, and linkage to bacterial resistance	Zhang QQ	Ying GG	Environmental Science and Technology	1903	2015
7	Emerging fungal threats to animal, plant and ecosystem health	Fisher MC	Gurr SJ	Nature	1674	2012
8	Diverse and abundant antibiotic resistance genes in Chinese swine farms	Zhu YG	Tiedje JM	Proceedings of the National Academy of Sciences of the United States of America	1376	2013
9	Global trends in antimicrobial use in food animals	Van Boeckel TP	Levin SA, Laxminarayan R	Proceedings of the National Academy of Sciences of the United States of America	1370	2015
10	Heavy use of prophylactic antibiotics in aquaculture: A growing problem for human and animal health and for the environment	Cabello FC	Cabello FC	Environmental Microbiology	1342	2006

### Analysis of Keywords

Keyword analysis was conducted on the four key topics of One Health, and keywords with a frequency >10 were extracted to generate a keyword co-occurrence map, which reflects the current direction of attention for each of the four topics. The size of the circle reflects the frequency of the keywords, and the thickness of the link represents the strength of the association. The following are the results of keyword analysis.

**Figure 5:**
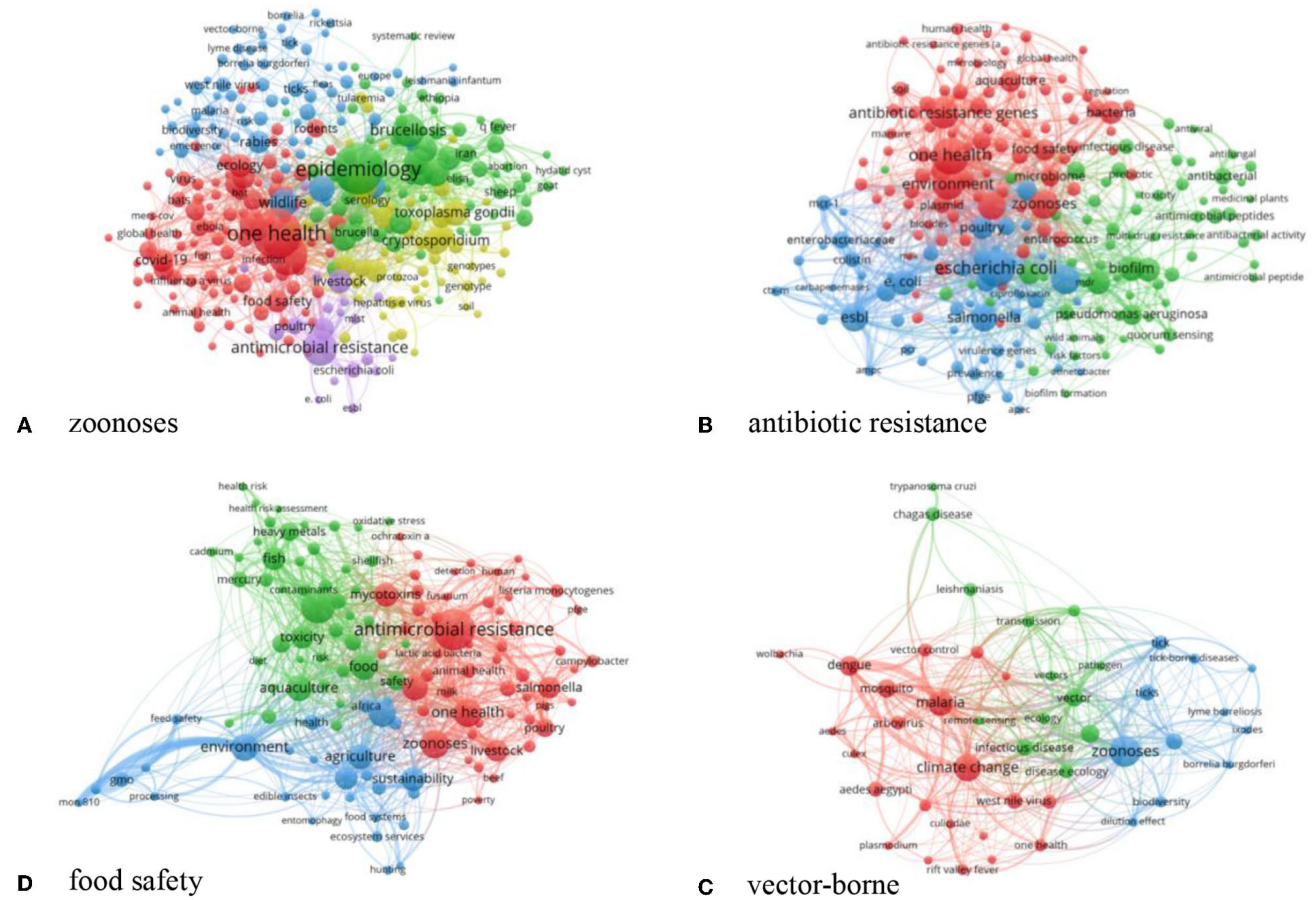
Co-occurrence clustering map of keywords on the four priority topics of One Health, 2003–2021.

Zoonoses are divided into five research hotspots (Figure 5A)- (1) viral zoonoses including COVID-19, Ebola hemorrhagic fever, and influenza (red area); (2) bacterial zoonoses such as brucellosis (green area); (3) parasitic zoonoses such as toxoplasmosis and cryptosporidiosis (yellow area); (4) vectors (blue area); and (5) antibiotic-resistant bacteria (purple area).

Antimicrobial resistance is divided into three research hotspots (Figure 5B)- (1) the detection of antimicrobial resistance genes (red area); (2) *Enterobacteriaceae* resistant bacteria such as *Escherichia coli* and *Salmonella* (blue area); and 3) methicillin-resistant *Staphylococcus aureus* and *Pseudomonas aeruginosa* (green area).

Food safety is divided into three research hotspots (Figure 5C)- (1) agricultural research related to the environment (blue area); (2) aquaculture research such as heavy metal contamination in aquatic plants and animals (green area); and (3) antimicrobial resistance in food (red area).

Vector-borne diseases is divided into three research hotspots (Figure 5D)- (1) mosquito-borne diseases (red area); (2) tick-borne diseases (blue area); and (3) vector transmission (green area).

## Discussion

This study used a bibliometric approach to examine the trajectory, bibliometric characteristics, and research hotspots of the four priority topics in One Health. The results show that the number of publications in these four areas has continued to grow since 2003, with acceleration starting in 2008. The US ranks in the top tier in One Health research with close international cross-institutional collaboration. Research disciplines dealing with One Health are mainly natural science disciplines, with less research conducted in social science disciplines. Keyword analysis reveals that the studies mainly focused on human and animal health, while less attention was paid to environmental health.

The number of publications is an important indicator in bibliometrics that reflects the amount of attention on a research field is receiving and whether the field is growing over time. This number has continued to grow in the field of One Health in recent years, with an acceleration starting in 2008. This is probably related to the multi-institutional collaboration between WHO, the World Organization for Animal Health (OIE), the Food and Agriculture Organization of the United Nations (FAO), the United Nations International Children's Emergency Fund (UNICEF), the World Bank, and the United Nations System Influenza Coordination (UNSIC) in 2008 (22). The steep rise in the number of publications after 2016 may have been influenced in part by the Zika and Ebola virus outbreaks (23, 24). The global spread of COVID-19 has also led the number of publications in One Health to take off again (25).

Zoonotic diseases have long been a key research area of One Health and its publications continue to grow, reaching a brief peak in 2020 possibly due to COVID-19 pandemic. The number of antimicrobial resistance-related publications has started to grow rapidly since 2014, which is likely spurred by the publication of WHO's first global report on antimicrobial resistance in 2014 based on data from 114 countries worldwide (26). The publications of food safety has started to emerge since 2018, while the vector-borne diseases topic have been studied mostly in humans and animals and less at the human-animal-environment interface.

The US, UK, and China have a relatively high number of publications on One Health. US and UK have established several One Health government agencies, international organizations, educational and research institutions, and research funds. UK and US universities are also actively collaborating on One Health training programs. However, most of the programs are established only for veterinary specialties (27). In China, there is no independent government agency on One Health. Few Chinese universities offer One Health specialties, and various departments and disciplines are still isolated from each other with little collaboration (7). In fact, many Chinese publications adopt a combined human-animal-environmental health approach without realizing that it is the application of the One Health approach.

Another interesting finding is that there is no correlation between Table 1 (Active journals in four priority areas of One Health research) and Table 3 (Top 10 cited One Health publications in the Scopus database). This is probably because most active journals in the One Health field are specialized journals that have a lower impact compared to top journals. It is quite understandable that in most cases, scholars are more willing to publish their work in a top journal rather than a good specialized one. Thus, most of the highly cited publications appear in top journals rather than specialized journals, even though the latter has a large number of publications. This situation may change if specialized journals that regularly publish articles in One Health reach top journal status or increase their impact factors.

The use of a multidisciplinary approach mentioned in the past definition of One Health has been expanded to a transdisciplinary approach in the latest CDC definition of One Health (16). Transdisciplinary research is defined as research efforts conducted by investigators from different disciplines working jointly to create new conceptual, theoretical, methodological, and translational innovations that integrate and move beyond discipline-specific approaches to address a common problem (28). The difference between multidisciplinarity and transdisciplinarity is that the former uses knowledge from different disciplines without going beyond their disciplinary boundaries, while the latter entails the integration of natural, social, and health sciences into a humanistic context and transcends their traditional disciplinary boundaries (29). However, according to the results of our analysis, the focus areas of One Health still revolve mainly around the natural sciences, while the social sciences are less involved, with 312 publications that account for merely one percent of the total number of publications between 2003 and 2021. Health policy support is one of the fundamental conditions for facilitating the initiation and successful implementation of One Health governance (30). Thus, social science disciplines such as politics, law, management, and economics should play an integral part in the transdisciplinary research on One Health for constructing practical policy recommendations. FAO, OIE, the United Nations Environment Programme (UNEP), and WHO jointly called on international experts to form the multidisciplinary OHHLEP on March 29, 2021. The OHHLEP will guide on matters related to One Health actions to support improved intergovernmental collaboration. It has an advisory role for its partners and is expected to provide evidence-based scientific and policy advice to address the challenges posed by One Health (9).

One Health represents a systematic approach that combines human, animal, and environmental health. However, our findings indicate that the current studies on One Health focus on human and animal health, while little attention is paid to environmental health. Meanwhile, due to the impact of COVID-19 pandemic, the tendency to concentrate on wildlife studies and neglect environmental health may be further reinforced. There is thus a need to fill the research gap between environmental health and human-animal health in order to improve the framework and implementation of One Health apporach (31).

Zoonoses with wild animals as hosts (mainly viral zoonoses) have increased in recent decades and focus on One Health research, while the role of One Health approach applied in parasitic diseases has also been discussed (32). Research on bacterial zoonoses is mainly associated with the discussion on increased antimicrobial resistance of bacteria. One Health is recognized as an effective approach to addressing health problems, including zoonoses, at the human-animal and environmental interfaces (16). There are examples around the world of applying One Health concepts to the prevention and control of zoonoses, including the Hendra Task Force in Australia (33) and the Sustainable One Health Program in Kenya (34). Moreover, in blocking imported infections from causing local transmissions of COVID-19, China has focused not only on human-to-human transmission but also on possible environmental transmission and the impact of animal vectors (35). Though many countries have now started to carry out zoonoses management through One Health, the actual implementation of the approaches varies greatly from country to country.

In terms of antimicrobial resistance, a bibliometric analysis pointed out that the most commonly studied pathogens were *Escherichia coli, Pseudomonas aeruginosa*, and *Staphylococcus aureus* in the order of decreasing frequency, and the detection of antimicrobial resistance genes was one of the hot spots, which is consistent with the results of this study (15). Drug-resistant bacteria in humans and animals can spread to each other through the food chain and water. To address the root cause of antimicrobial resistance, efforts have been made in the prevention and control of cross contamination through the food chain and water (36). In 2015, WHO pointed out in the Global Action Plan for Antimicrobial Resistance report that antimicrobial resistance primarily affects human health, but that the factors contributing to antimicrobial resistance and its consequences (economic and otherwise) surpass the domain of health (37). Thus, they argue that a “One Health” mindset and beyond, with a coherent, comprehensive, and integrated approach at global, regional, and national levels that involves different sectors and actors such as human medicine, veterinary medicine, agriculture, finance, environment, and consumers is required (37).

The widespread use of antibiotics in agriculture and aquaculture as growth promoters of food animals and fish has caused and exacerbated the spread of antimicrobial resistance (38). Foodborne diseases occur frequently not only in developing countries but also in developed countries and are caused by the consumption of food contaminated with various microorganisms (39). According to the WHO, there are more than 200 foodborne diseases (40). FAO, OIE, and WHO recognized that addressing health risks at the human-animal-environment interface requires a strong partnership among all actors involved (41). Institute of Medicine (IOM) has organized a One Health workshop on food safety to encourage countries to disclose food safety issues and investigation records and to involve various organizations and sectors with the common goal of promoting food safety (42).

Vector-borne diseases are increasingly threatening animal and human health in both developing and developed countries. An increasing amount of data suggests that this upward trend will continue (43). Human pets such as dogs and cats are potential hosts for vector-borne diseases. Thus, identifying and controlling vector-borne diseases in pet hosts is one of the major challenges in addressing vector-borne diseases using the One Health approach (44). Past studies have proven that mosquitoes or ticks are the most important vectors of pathogens for animals and humans (45). Therefore, disease vector control is a priority from both veterinary and public health perspectives (43).

Although not included in our analysis, climate change has been the subject of global attention in recent years. However, the focus is often limited to the impacts of climate change on human health, neglecting the important impacts on animal and environmental health. Climate change affects many sectors. This means that a collaborative, cross-sectoral, and interdisciplinary approach to One Health is essential to address the challenges posed by climate change (46). Climate change is currently driving a general redistribution of life on earth that could lead to new biomes and rapid changes in ecosystem functions, the consequences of which could propagate and affect biomes and human communities (47). In addition, climate change can influence the spread and persistence of infectious diseases (48). For example, studies have found that average temperature, minimum temperature, and air quality are strongly associated with the COVID-19 pandemic (49). In addition, factors affecting the environment include depletion of forests, expansion and modernization of agricultural practices, and natural disasters such as floods. All of these may lead to changes in microbial ecological niches and promote microbial adaptation to human hosts (50). For example, deforestation in the Amazon region has created breeding sites for *Anopheles darlingi*, the main malaria vector in Brazil, and forest fires and road construction have increased the risk of malaria transmission there (51). Therefore, the scientific community should also focus on research on climate change at the human-animal-environment interface.

This study has some limitations. First, although this study analyzed as many keywords as possible, the selection and classification were to some extent subjective, which could lead to literatures' selection bias. Second, when using VOSviewer for keyword clustering analysis, the interpretation of cluster names may not be precise enough for conducting research hotspot analysis due to the software algorithm and subjective judgment. Third, this study analyzed the content of only four topics in the field of One Health. Thus, it is not representative of the entire One Health field. Last, although the Scopus database contains a more comprehensive range of publications and is the primary source of data for bibliometric analysis, there is a risk of losing suitable publications in other databases that are not included in Scopus.

## Conclusions

This study assessed and analyzed publications on four priority topics in the field of One Health—zoonotic diseases, antimicrobial resistance, food safety, and vector-borne infections. Research in this area has evolved rapidly over the past decade or so. With the outbreak or spread of emerging and re-emerging infectious diseases (e.g., COVID-19), more and more research in this emerging field has shifted focus to the application of One Health approaches at the human-animal-environment interface to address complex global public health problems. In order to generate an integrated solution to challenges posed collectively on humans, animals, and the environment, it is necessary to promote cross-sectoral, trans-disciplinary, and cross-regional cooperation and advocate an integrated human-animal-environment management strategy. Finally, as most of the disciplines involved in One Health with medicine-related issues only, there should be more interdisciplinary exchanges with non-medical disciplines such as politics, law, economics, and management.

## Author Contributions

LM contributed to the study design, manuscript writing, literature search, analysis, and visualization. HL, WD, DW, XG, and XZ contributed to draft review, editing, and validation. SP and SL participated in language editing. All authors contributed to manuscript revision, read, and approved the submitted version.

## Funding

This study was supported by China Medical Board [Grant No: 20-365] and the programme of the Chinese Center for Tropical Diseases Research [Grant No: 131031104000160004].

## Conflict of Interest

The authors declare that the research was conducted in the absence of any commercial or financial relationships that could be construed as a potential conflict of interest.

## Publisher's Note

All claims expressed in this article are solely those of the authors and do not necessarily represent those of their affiliated organizations, or those of the publisher, the editors and the reviewers. Any product that may be evaluated in this article, or claim that may be made by its manufacturer, is not guaranteed or endorsed by the publisher.
